# Relationship between the novel and traditional anthropometric indices and subclinical atherosclerosis evaluated by carotid intima-media thickness (c-IMT)

**DOI:** 10.3389/fnut.2023.1170450

**Published:** 2023-06-30

**Authors:** Clara Costo-Muriel, Julián F. Calderón-García, Sergio Rico-Martín, Javier Galán-González, Guillermo Escudero-Sánchez, Carmen Sánchez-Bacaicoa, Francisco J. Rodríguez-Velasco, Esperanza Santano-Mogena, César Fonseca, Juan F. Sánchez Muñoz-Torrero

**Affiliations:** ^1^Department of Internal Medicine, Hospital Comarcal de la Axarquía, Málaga, Spain; ^2^Department of Nursing, Nursing and Occupational Therapy College, University of Extremadura, Cáceres, Spain; ^3^Department of Internal Medicine, Hospital San Pedro Alcántara, Cáceres, Spain; ^4^Department of Internal Medicine, Hospital Virgen Del Puerto, Plasencia, Cáceres, Spain; ^5^Department of Family Medicine, Hospital of Menorca, Minorca, Spain; ^6^Department of Nursing, Faculty of Medicine and Health Science, University of Extremadura, Badajoz, Spain; ^7^Department of Nursing, University of Évora, Evora, Portugal; ^8^Department of Nursing, Comprehensive Health Research Centre (CHRC), Evora, Portugal

**Keywords:** anthropometric indices, carotid intima-media thickness, cardiovascular risk factors, subclinical atherosclerosis, carotid ultrasonography

## Abstract

**Introduction:**

Over the last few years, novel anthropometric indices have been developed as an alternative to body mass index (BMI) and other traditional anthropometric measurements to enhance the estimate of fat proportion and its relationship to a future cardiovascular event. The purpose of this study was to investigate the association of carotid intima-media thickness (c-IMT) estimated by Doppler ultrasound with current anthropometric indices (traditional and novel).

**Methods:**

A cross-sectional study was conducted on a total of 789 Spanish patients. Traditional (BMI, WHR, and WHtR) and new (WWI, AVI, ABSI, BRI, BAI, CUN-BAE, and CI) anthropometric indices were determined, and carotid Doppler ultrasound was performed to evaluate c-IMT (≥0.90 mm).

**Results:**

Most of the anthropometric indices analyzed were significantly higher among patients with pathological c-IMT, except for BMI, BAI, and CUN-BAE. In multiple linear regression analysis, c-IMT was positively related to ABSI, AVI, BRI, CI, and WWI but not to CUN-BAE, BAI, or traditional anthropometric indices. Similarly, in univariate analysis, all indices were associated with a c-IMT of ≥0.90 mm (*p* < 0.05), except BMI, BAI, and CUN-BAE; however, only ABSI (adjusted OR: 1.61; 95% CI: 1.08–2.40; *p* = 0.017), CI (adjusted OR: 1.73; 95% CI: 1.15–2.60; *p* = 0.008), and WWI (adjusted OR: 1.74; 95% CI: 1.14–2.64; *p* = 0.009) were significantly associated in multivariate analysis. Finally, CI, ABSI, and WWI provided the largest AUC, and BMI and CUN-BAE showed the lowest AUC.

**Conclusion:**

ABSI, CI, and WWI were positively associated with pathological c-IMT (≥0.90 mm), independent of other confounders.

## Introduction

Despite improvements in recent years, cardiovascular (CV) diseases remain to be the main cause of death worldwide (1, 2). Atherosclerotic injuries are the common cause of all of these diseases. The lesions are involved in a complex chronic degenerative process that evolves over years and occurs at the level of the arterial intimal layer, resulting in progressive asymmetric focal thickening composed of connective tissue elements, lipids, and debris (3). Detection of atherosclerosis in asymptomatic individuals using different strategies helps us to predict and thus prevent future CV events (4).

Carotid ultrasound, including carotid intima-media thickness (c-IMT) ultrasound, has been suggested as a potential technique to assist CV risk stratification, as it is a safe, low-cost, and commonly available method that directly evaluates arterial atherosclerosis (5–7). In multiple previous studies, c-IMT has been demonstrated to be associated with the incidence of CV events and CV risk factors (8–12).

On the other hand, anthropometric indices are accepted as low-cost, simple, and non-invasive methods for population screening and early identification of obesity. Body mass index (BMI), waist circumference (WC), waist-to-height ratio (WHtR), and waist-to-hip ratio (WHR) have always been the most frequently used in daily clinical practice (13). In the last few years, novel anthropometric indices have been developed as an alternative to traditional anthropometric measurements to enhance the estimation of fat content and its relationship to CV risk (14).

A body shape index (ABSI) assesses general and visceral adiposity and is better related to abdominal fat than BMI (15). The body roundness index (BRI) predicts the amount of total and regional fat and is considered a predictor of metabolic syndrome in heterogeneous populations, being superior to BMI in numerous studies (16). The body adiposity index (BAI) is derived from hip circumference and height to calculate the amount of corporal adiposity (17). The Clínica Universidad de Navarra-Body Adiposity Estimator (CUN-BAE) equation is an estimator of the fat content with age, sex, and BMI and has been proven to be useful for selecting patients at high metabolic risk (18). The abdominal volume index (AVI) measures the volume of abdominal fat volume and presents a positive association with metabolic syndrome (19). The weight-adjusted waist index (WWI) has shown an association with CV morbidity and mortality (20). The conicity index (CI) uses weight, height, and abdominal circumference variables to estimate the degree of obesity and fat distribution (21).

Each of the novel anthropometric measures has been related to CV risk factors, though more investigation is needed to establish their precision and accuracy (14). The present study aimed to evaluate the possible association between novel and traditional anthropometric measures and subclinical carotid atherosclerosis assessed by c-IMT measured with Doppler ultrasound.

## Methodology

### Study population and design

**Figure 1:**
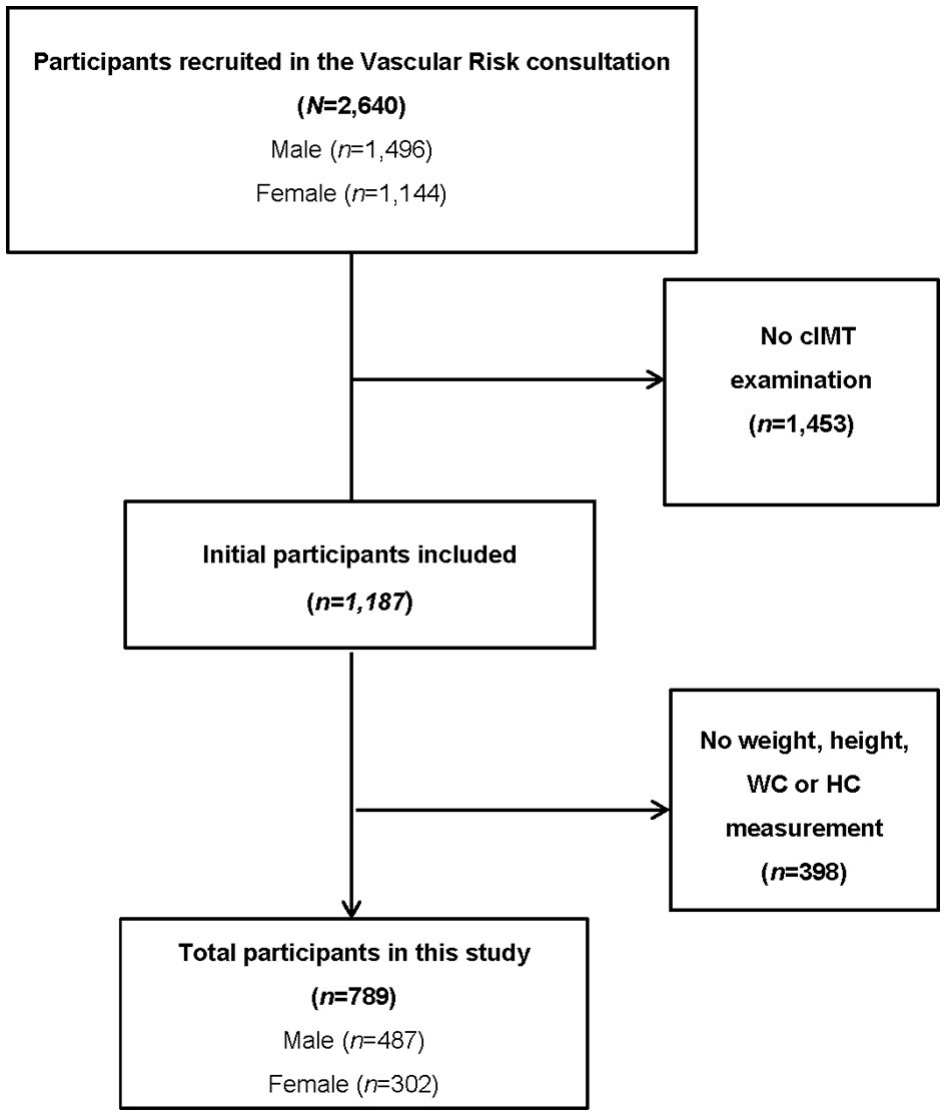
Participants selection process.

A cross-sectional study was carried out between June 2021 and September 2022, involving 789 subjects who consecutively attended the Vascular Risk consultation of the Grupo de Estudios de Enfermedades VASculares (GEEVAS) research group of Cáceres (Spain). The participant selection process is shown in Figure 1. Initially, 2,640 subjects were screened (of whom, 1,453 were excluded due to failure to complete the c-IMT assessment). A total of 1,187 participants were recruited, although 398 were excluded because no anthropometric measurement was performed. Finally, 789 patients aged between 18 and 80 were studied. Participants with anatomical disorders that made cervical examination difficult, institutionalized patients, chronic kidney disease in dialysis replacement treatment patients, pregnant women, mentally ill or incapacitated patients, and those who had been diagnosed with terminal diseases were excluded. Each participant gave written informed consent to participate in the study. The study protocol was in accordance with the Declaration of Helsinki and was previously approved by the Ethics Committee (Ref. 047-2021) of the University Hospital “San Pedro de Alcántara” of Cáceres (Spain).

### Study variables

Clinical variables associated with CV risk (age, smoking status, sedentary lifestyle, presence of dyslipidemia, hypertension, diabetes mellitus, metabolic syndrome, and obesity), current medical treatment, and previous CV events were extracted from the patient's medical history. After a period longer than 10 h of fasting, a blood test was performed including coagulation, biochemistry with hepatic and renal function, a complete blood count, and glycaemic and lipid profiling.

Each subject completed the physical assessment. Blood pressure (BP) readings were taken in the first hour in the morning with the patient relaxed and seated in accordance with the latest recommendations of the European Society of Hypertension (22). A total of three measurements of systolic (SBP) and diastolic blood pressure (DBP) were performed, and their values were estimated as the average values of the last two readings taken with an oscillometric device (OMRON model HEM-907). Consequently, pulse pressure (PP) was determined: PP = SBP-DBP.

Weight was measured with a precision biomedical scale and the height was determined with a Harpenden stadiometer. Both measures were carried out with participants wearing light clothing and no shoes. A non-elastic tape was used to assess the hip circumference (HC) and WC following the Spanish Society for the Study of Obesity recommendations (23). Novel and traditional anthropometric indices were calculated using the formulas shown in Table 1.

**Table 1:** Formulas to calculate traditional and novel anthropometric indices and cut-off considered in this study.

**Anthropometric indices**	**Formula**	**Cut-off**
**Traditional anthropometric indices**		
BMI	weight (kg)/height^2^ (m)	30 ≥ kg/m^2^
WHR	WC (cm)/HC (cm)	> 0.85 women; > 0.94 men
WHtR	WC (cm)/height (cm)	> 0.5
**Novel anthropometric indices**		
ABSI	WC (m)/(BMI^2/3^(kg/m^2^) · Height^1/2^ (m))	≥0.00866^a^
AVI	(2 · WC^2^ (cm) + 0.7(WC – HC)^2^ (cm)/1000	≥23.89 ^a^
BAI	BAI = [HC (m)/Height^2/3^(m)] – 18	≥ 36.40 ^a^
BRI	364.2–365.5 [1- π^−2^ WC^2^ (m) · Height^−2^ (m)]^1/2^	≥ 6.96 ^a^
CI	0.109^−1^ WC (m) [Weight (kg)/Height (m)]^−1/2^	≥ 1.39 ^a^
CUN-BAE	(3.1723 · BMI) – (0.026 · BMI^2^) + (0.181· BMI · sex) – (0.02 · BMI · age) – (0.005 · BMI^2^ · sex) + (0.00021 · BMI^2^ · age)	≥ 41.70 ^a^
WWI	WC/$\sqrt{weight}$	≥ 11.92 ^a^

a Due to the non-existence of cutoff points for the new anthropometric indices, the highest quartile values were considered.

ABSI, a body shape index; AVI, abdominal volume index; BAI, body adiposity index; BMI, body mass index; BRI, body roundness index; CI, conicity index; CUN-BAE, Clínica Universidad de Navarra-Body Adiposity Estimator; HC: Hip circumference; WC: Waist circumference; WHR, waist-to-hip ratio; WHtR, waist-to-height ratio; WWI, weigh adjusted waist index.

### Carotid ultrasonography assessment (c-IMT)

Carotid ultrasonography was carried out according to the Mannheim Consensus recommendations (24) by a single well-trained operator. A LOGIQ S7^®^ ultrasound device (General Electric Healthcare, United Kingdom) with an 8-MHz linear probe was used. The c-IMT was determined automatically with the specific software included in the ultrasound device. All the measurement was performed with the patient in the supine position, the head lightly rotated away from the carotid artery was assessed, and the neck hyperextended. A mean c-IMT of ≥0.90 mm was considered pathological (carotid subclinical atherosclerosis) (25).

### Statistical analysis

Categorical variables are presented as frequencies (%) and continuous variables are presented as the averages ± standard deviations (sd). Subjects were compared according to the presence or absence of pathological c-IMT. The distribution was considered normal when a *p* > 0.05 was found in the Kolmogorov–Smirnov test. Categorical variables were compared utilizing the χ2 test or Fisher's exact test when in any of the groups, the observed frequency was <5. Moreover, the Student's *t*-test (if normal distribution) or the Mann–Whitney U-test (if non-normal distribution) was used to compare continuous variables. Pearson's correlations were utilized to investigate the relation between c-IMT and the novel and traditional anthropometric measures. Multiple linear regression applying the enter method was employed to assess whether the studied variables were predictive of mean c-IMT.

In addition, uni- and multivariate logistic regression analyses were carried out to assess the association between dependent (pathological c-IMT) and independent variables. Due to the lack of cutoff points for the novel anthropometric measures, the values of the greatest quartile were considered while for traditional anthropometric measures, previously published pathological cutoff points were assumed. Table 1 shows the pathological cutoff used in this study. Odds ratios (ORs) and subsequent 95% confidence intervals (CIs) were analyzed. When the independent variables had a *p* < 0.10 in the univariate analysis, they were incorporated into the multivariate analysis.

Finally, the predictive performance of anthropometric indices for identifying carotid subclinical atherosclerosis (c-IMT ≥ 0.90 mm) was analyzed by receiver operating characteristic (ROC) analysis. The optimal cutoff values of anthropometric indices to detect pathological c-IMT (≥0.90 mm) from ROC analyses were determined by the maximum Youden's index (sensitivity + specificity −1).

Statistical analysis was carried out using IBM SPSS V.27 software (IBM Corporation, Armonk, NY, USA).

## Results

A total of 789 (61.8% men) patients with an average age of 64.14 ± 12.20 years were analyzed. Of these, 195 had a c-IMT of ≥ 0.90 mm. The variables were compared between patients with non-pathological c-IMT (<0.90 mm) and patients with pathological c-IMT (≥ 0.90 mm) (Table 2). Significant differences were observed in favor of subjects with a c-IMT ≥ 0.90 mm in terms of age, male sex, diabetes, hypertension, metabolic syndrome, and CV events. Smoking, dyslipidemia, obesity, and a sedentary lifestyle were not significant. Patients with pathological c-IMT (≥ 0.90 mm) had significantly higher values of systolic and diastolic blood pressure, pulse pressure, fast plasma glucose, glycated hemoglobin, and creatine clearance. The use of antidiabetic and antihypertensive treatments was significantly greater. However, values of total cholesterol and low-density lipoprotein cholesterol were significantly lower in patients with c-IMT≥ 0.90 mm. With respect to the anthropometric indices analyzed, the ABSI, AVI, BRI, WWI, WHR, and WHtR were significantly higher among patients with pathological c-IMT (≥ 0.90 mm). However, non-significant differences were found with respect to BMI, BAI, and CUN-BAE.

**Table 2:** Baseline characteristics among patients according to the presence of pathological c-IMT (≥0.90 mm).

	**Subjects with c-IMT < 0.90 mm (*n* = 594)**	**Subjects with c-IMT≥0.90 mm (*n* = 195)**	***p*-value**
Age (years)	61.82 ± 11.96	71.20 ± 10.05	**< 0.001**
Gender-men (%)	345 (58.2%)	142 (72.8%)	**< 0.001**
**CV Risk factors**			
Non-smokers (%)	192 (32.4%)	55 (28.2%)	0.276
Hypertension (%)	342 (57.7%)	161 (82.6%)	**< 0.001**
Dyslipemia (%)	518 (87.4%)	165 (84.6%)	0.329
Diabetes (%)	161 (27.2%)	91 (46.7%)	**< 0.001**
Obesity (%)	238 (40.1%)	82 (42.1%)	0.553
Metabolic Syndrome (%)	192 (32.4%)	116 (59.5%)	**< 0.001**
Sedentary (%)	211 (35.6%)	66 (33.8%)	0.660
CV event (%)	123 (20.7%)	87 (44.6%)	**< 0.001**
**Clinical and laboratory evaluation**			
SBP (mmHg)	138.01 ± 18.05	143.55 ± 19.33	**< 0.001**
DBP (mmHg)	81.43 ± 10.04	77.80 ± 10.87	**< 0.001**
PP (mmHg)	56.57 ± 16.60	65.74 ± 17.26	**< 0.001**
Total cholesterol (mg/dL)	176.85 ± 42.46	163.92 ± 35.52	**< 0.001**
HDL (mg/dL)	51.52 ± 15.94	50.12 ± 15.94	0.279
LDL (mg/dL)	97.93 ± 36.30	87.93 ± 31.93	**0.001**
Triglyceride (mg/dL)	145.24 ± 104.55	140.01 ± 87.22	0.529
FPG (mg/dL)	108.71 ± 29.09	115.77 ± 33.53	**0.009**
HbA1C (%)	6.03 ± 0.96	6.32 ± 1.14	**0.002**
eGFR (ml/min)	111.43 ± 70.83	97.07 ± 93.38	**0.027**
**Drugs**			
Antihypertensive drugs (%)	19 (8.3%)	176 (31.4%)	**< 0.001**
Lipid lowering drugs (%)	26 (21.1%)	169 (25.3%)	0.321
Antidiabetic drugs (%)	94 (19.8%)	101 (32.0%)	**< 0.001**
**Traditional anthropometric indices**			
BMI (Kg/m^2^)	29.47 ± 5.12	29.59 ± 4.54	0.758
WHR	0.93 ± 0.093	0.96 ± 0.102	**< 0.001**
WHtR	0.60 ± 0.08	0.63 ± 0.08	**< 0.001**
**Novel anthropometric indices**			
ABSI	0.0816 ± 0.007	0.085 ± 0.008	**< 0.001**
AVI	20.28 ± 5.55	22.48 ± 6.35	**< 0.001**
BAI	32.73 ± 6.89	33.51 ± 6.15	0.159
BRI	5.67 ± 1.99	6.51 ± 2.09	**< 0.001**
CI	1.31 ± 0.11	1.38 ± 0.13	**< 0.001**
CUN-BAE	36.25 ± 7.99	35.82 ± 7.08	0.475
WWI	11.16 ± 1.03	11.80 ± 1.15	**< 0.001**

ABSI, a body shape index; AVI, abdominal volume index; BAI, body adiposity index; BMI, body mass index; BRI, body roundness index; CAD, coronary arterial disease; CI, conicity index; c-IMT, carotid intima media thickness; CUN-BAE, Clínica Universidad de Navarra-Body Adiposity Estimator; CV, cardiovascular; DBP: Diastolic Blood Pressure; FPG, fast plasma glucosa; HbA1c, glycated hemoglobin; HDL, high-density lipoprotein; LDL, low-density lipoprotein; PP, pulse pressure; SBP, systolic blood pressure; WHR, waist-to-hip ratio; WHtR, waist-to-height ratio; WWI, weigh adjusted waist index. Bold means statistically significant values.

On the other hand, c-IMT was significantly correlated with all anthropometric indices studied, except for CUN-BAE (Table 3). We further investigated the possible significant relationship of c-IMT with the anthropometric indices studied by multiple linear regression. This analysis was adjusted for confounding variables. C-IMT was positively related (*p* < 0.05) to ABSI, AVI, BRI, CI, and WWI but not to CUN-BAE, BAI, or the traditional anthropometric indices.

**Table 3:** Correlation and multiple linear regression analysis between c-IMT and anthropometric indices.

	**Correlation analysis**		**Multiple linear regression analysis**				
	**R**	***p*** **-value**	**Model R** ^2^	**Model Adjusted R** ^2^	**Standardized** β	***t***	***p*** **-value**
**Traditional anthropometric indices**							
BMI (Kg/m^2^)	0.106	**0.003**	0.360	0.354	0.023	0.730	0.466
WHR	0.226	**< 0.001**	0.360	0.354	0.051	1.479	0.140
WHtR	0.266	**< 0.001**	0.360	0.354	0.046	1.388	0.166
**Novel anthropometric indices**							
ABSI	0.289	**< 0.001**	0.362	0.356	0.079	2.381	**0.018**
AVI	0.250	**< 0.001**	0.365	0.358	0.080	2.514	**0.016**
BAI	0.122	**0.001**	0.360	0.354	0.013	0.324	0.746
BRI	0.279	**< 0.001**	0.365	0.358	0.077	2.316	**0.021**
CI	0.321	**< 0.001**	0.363	0.357	0.089	2.625	**0.009**
CUN-BAE	0.043	0.227	0.360	0.354	0.043	0.903	0.400
WWI	0.334	**< 0.001**	0.367	0.360	0.093	2.701	**0.007**

Multiple linear regression analysis adjusted by age, sex, SBP, DBP, PP, total cholesterol, HDL, LDL, triglycerides, FPG, HbA1C, and eGFR.

ABSI, a body shape index; AVI, abdominal volume index; BAI, body adiposity index; BMI, body mass index; BRI, body roundness index; CI, conicity index; CUN-BAE, Clínica Universidad de Navarra-Body Adiposity Estimator; WHR, waist-to-hip ratio; WHtR, waist-to-height ratio; WWI, weigh adjusted waist index. Bold means statistically significant values.

In the univariate analysis (Table 4), the variables that were significantly associated with a c-IMT ≥ 0.90 mm were male sex, age ≥ 65 years, diabetes, hypertension, metabolic syndrome, cardiovascular event, eGFR < 60 ml/min, FPG ≥ 126 mg/dL, HbA1C ≥ 6.5%, TC ≥ 190 mg/dL, LDL ≥ 100 mg/dL, SBP ≥140 mmHg, DBP ≥ 90 mmHg, and PP ≥ 60 mmHg.

**Table 4:** Predictors of pathological c-IMT (≥0.90 mm). Univariate analysis.

	**Subjects with c-IMT≥0.90 mm (*n* = 195) OR (CI%95)**	***p*-value**
Age (years) ≥ 65	4.41 (3.07–6.34)	**< 0.001**
Men (%)	1.92 (1.35–2.74)	**< 0.001**
Non–smokers	0.82 (0.57–1.17)	0.276
Current Smokers (%)	1.00 (0.64–1.55)	0.986
Ex–smokers (%)	1.18 (0.85–1.63)	0.318
Hypertension (%)	3.47 (2.32–5.20)	**< 0.001**
Dyslipidemia (%)	0.49 (0.50–1.25)	0.329
Diabetes (%)	2.34 (1.68–3.28)	**< 0.001**
Obesity (%)	1.10 (0.79–1.52)	0.553
Sedentary (%)	0.92 (0.65–1.30)	0.660
Metabolic Syndrome (%)	3.06 (2.19–4.28)	**< 0.001**
CV event (%)	3.08 (2.18–4.34)	**< 0.001**
SBP (mmHg) ≥ 140	1.77 (1.27–2.46)	**0.001**
DBP (mmHg) ≥ 90	0.55 (0.35–0.85)	**0.008**
PP (mmHg) ≥ 60	3.19 (2.26–4.49)	**< 0.001**
TC (mg/dL) ≥ 190	0.57 (0.39–0.83)	**0.004**
LDL (mg/dL) ≥ 100	0.52 (0.37–0.74)	**< 0.001**
Triglyceride (mg/dL) ≥ 200	0.82 (0.52–1.30)	0.416
FPG (mg/dL) ≥ 126	1.63 (1.11–2.40)	**0.013**
HbA1C (%) ≥ 6.5	1.63 (1.11–2.40)	**0.011**
eGFR (ml/min) < 60	1.54 (1.06–2.25)	**0.023**
Antihypertensive drugs	5.09 (3.08–8.41)	**< 0.001**
Lipid lowering drugs	1.26 (0.79–2.01)	0.321
Antidiabetic drugs	1.89 (1.36–2.63)	**< 0.001**

CV, cardiovascular; DBP: Diastolic Blood Pressure; FPG, fast plasma glucose; HbA1c, glycated hemoglobin; HDL, high–density lipoprotein; LDL, low–density lipoprotein; PP, pulse pressure; SBP, systolic blood pressure; TC, total cholesterol. Bold means statistically significant values.

In the univariate analysis of anthropometric indices (Table 5), WHR (OR: 1.64; 95% CI: 1.13–2.40; *p* = 0.009), WHtR (OR: 2.47; 95% CI: 1.10–5.54; *p* = 0.028), ABSI (OR: 2.66; 95% CI: 1.88–3.75; *p* < 0.001), AVI (OR: 1.51; 95% CI: 1.08–2.12; *p* = 0.015), BRI (OR: 1.93; 95% CI: 1.36–2.73; *p* < 0.001), CI (OR: 3.07; 95% CI: 2.18–4.34; *p* < 0.001), and WWI (OR: 2.77; 95% CI: 1.96–3.91; *p* < 0.001) presented a significantly positive association with a c–IMT of ≥ 0.90 mm. However, only ABSI (adjusted OR: 1.61; 95% CI: 1.08–2.40; *p* = 0.017), CI (adjusted OR: 1.73; 95% CI: 1.15–2.60; *p* = 0.008), and WWI (adjusted OR: 1.74; 95% CI: 1.14–2.64; *p* = 0.009) were significant in the multivariate analysis.

**Table 5:** Predictors of pathological c–IMT (≥0.90 mm). Univariate and Multivariable analyses.

	**Univariate**		**Multivariable**	
	**OR (CI%95)**	***p*** **–value**	**OR (CI%95)**	***p*** **–value**
**Traditional anthropometric indices**				
BMI ≥ 30 Kg/m^2^	1.10 (0.79–1.52)	0.553	–	–
WHR > 0.85 in women or 0.94 in men	1.64 (1.13–2.40)	**0.009**	0.95 (0.60–1.50)	0.840
WHtR > 0.5	2.47 (1.10–5.54)	**0.028**	0.77 (0.28–2.08)	0.608
**Novel anthropometric indices**				
ABSI ≥ 0.0866	2.66 (1.88–3.75)	**< 0.001**	1.61 (1.08–2.40)	**0.017**
AVI ≥ 23.89	1.51 (1.08–2.12)	**0.015**	0.94 (0.62–1.43)	0.797
BAI ≥ 36.40	1.29 (0.89–1.85)	0.168	-	-
BRI ≥ 6.96	1.93 (1.36–2.73)	**< 0.001**	1.30 (0.85–1.97)	0.215
CI ≥ 1.39	3.07 (2.18–4.34)	**< 0.001**	1.73 (1.15–2.60)	**0.008**
CUN–BAE ≥ 41.70	0.75 (0.51–1.10)	0.144	-	-
WWI ≥ 11.92	2.77 (1.96–3.91)	**< 0.001**	1.74 (1.14–2.64)	**0.009**

Multivariate analysis was adjusted by age ≥ 65 years, male gender, presence of arterial hypertension, diabetes mellitus, metabolic syndrome, cardiovascular event, SBP ≥140 mmHg, DBP ≥ 90 mmHg, PP ≥ 60 mmHg, TC ≥ 190 mg/dL, LDL ≥ 100 mg/dL, FPG ≥ 126 mg/dL, HbA1C ≥ 6.5%, eGFR < 60 ml/min, and use of antidiabetic and antihypertensive drugs.

ABSI, a body shape index; AVI, abdominal volume index; BAI, body adiposity index; BMI, body mass index; BRI, body roundness index; CI, conicity index; CUN-BAE, Clínica Universidad de Navarra-Body Adiposity Estimator; WHR, waist-to-hip ratio; WHtR, waist-to-height ratio; WWI, weigh adjusted waist index. Bold means statistically significant values.

**Figure 2:**
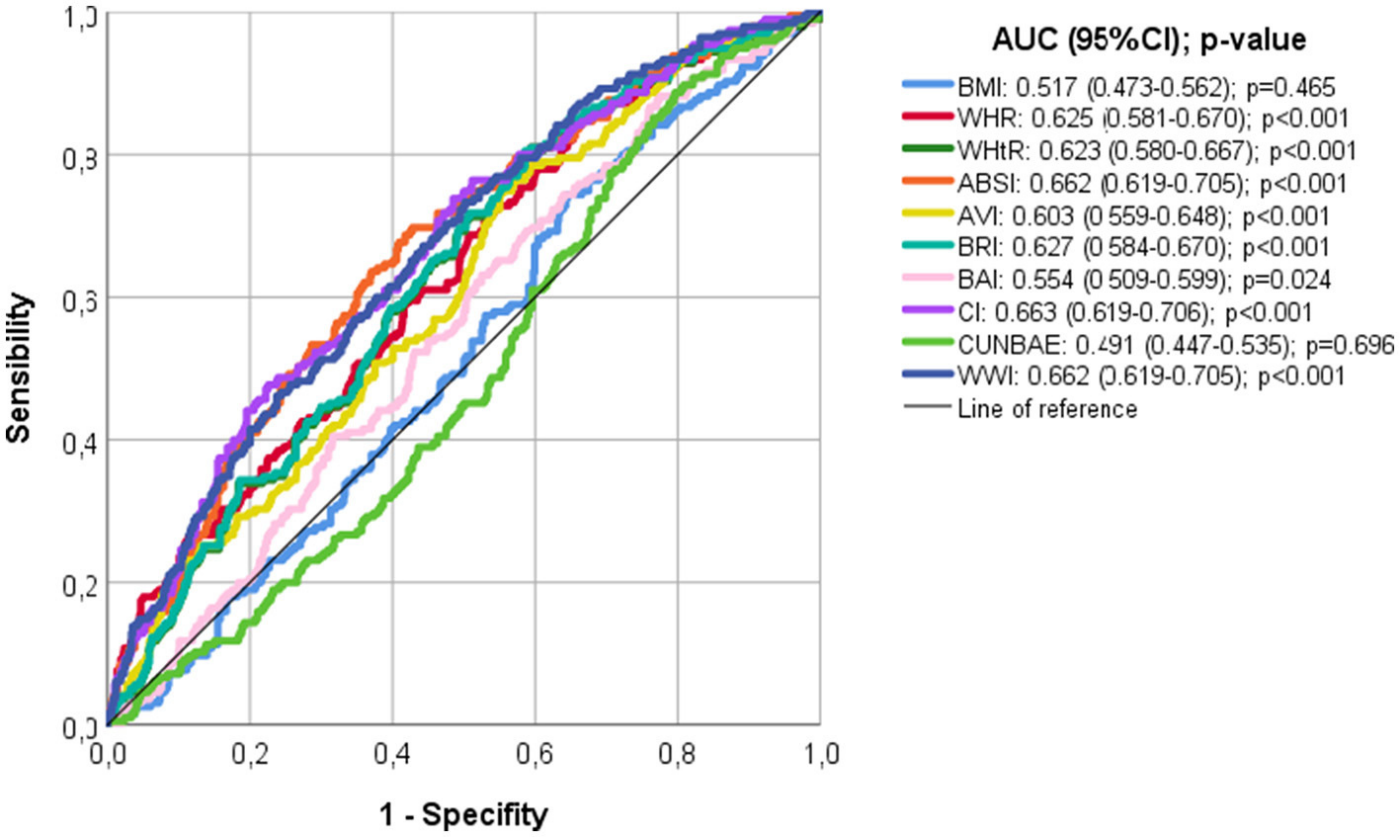
ROC analysis to predict pathological c-IMT (≥0.90 mm).

Finally, according to the ROC analyses (Figure 2), CI, ABSI, and WWI showed the largest area under the curve (AUC: 0.663, 0.662, and 0.663, respectively), and BMI and CUN-BAE showed the smallest AUC (0.491 and 0.517, respectively). The optimal cut-off values to detect pathological c-IMT (>0.9 mm) from ROC analyses were 0.0824 for ABSI, 1.31 for CI, and 11.07 for WWI.

## Discussion

In this cross-sectional study that included 789 Spanish subjects, ABSI, CI, and WWI were significantly associated with subclinical carotid atherosclerosis assessed by c-IMT measured with Doppler ultrasound after multivariate adjustment.

In multiple previous studies, increased c-IMT has been associated with CV risk factors and the occurrence of CV events (9–11). Furthermore, increased c-IMT has been associated with atherosclerosis in different arterial beds, involvement of other systemic organs (11), and prediction of future CV events (4). Similarly, the carotid plaque has been demonstrated to be related to CV risk factors (26) and outperform c-IMT in predicting myocardial infarction (5), being a good prognostic marker of future CV events (27). In the latest 2019 European Society of Cardiology and European Atherosclerosis Society guidelines for the management of dyslipidemias (28), assessment of carotid atherosclerotic burden by ultrasound has a predictive value for CV events comparable to that of coronary calcium measurement by computed tomography (29–31); in contrast, c-IMT measurement is inferior to carotid plaque detection (32, 33).

Some studies support that c-IMT and carotid plaque represent different phases of atherogenesis, such that the progressive increase in c-IMT (wall growth) would be a state before the development of carotid plaque (34–37). According to this theory, c-IMT would be a predictor of the formation of new plaques and would highlight its importance as a tool for detecting individuals at high risk for future CVD in its early stages. On the other hand, other studies argue that c-IMT and carotid plaque represent two different phases of vascular remodeling (38, 39), supported by genetic studies that have revealed the existence of different genes associated with c-IMT and carotid plaque (40, 41).

Following this controversy, a meta-analysis was performed in 2019 of seven cohort studies including a total of 9,341 subjects who showed a significant relationship between the occurrence of first carotid plaque and increased baseline c-IMT, although this would not necessarily involve a direct association between the two. There is a possibility that the considerable number of risk factors they share may have played a role, despite multivariate adjustment (7). It is clear that both measurements have been shown to be associated with CV risk factors and with the incidence of CV events, with the union of both measures being a better predictor of future CV events than separately (5, 42, 43).

On the other hand, obesity, which is considered a public health problem, is a chronic disease linked with an increased risk of CV disease and all-cause mortality (44–46). Currently, scientific societies recommend BMI and WC as indicators of obesity (13, 47, 48). However, these measures have several limitations, as they neither discriminate between fat and muscle mass nor do they take into account fat distribution, which is one of the main predictors of metabolic disease risk (18, 49). In recent years, new anthropometric indices have emerged with the aim of improving the estimation of fat proportion and its relation with CV risk (14).

In our study, all anthropometric indices, except BMI, CUN-BAE, and BAI, showed an association with pathological c-IMT in the univariate analysis, but only ABSI, CI, and WWI did so after accounting for confounders in the multivariate analysis. Recently, our research group has published the relationship between subclinical atherosclerosis in this study population assessed by the presence of carotid plaques, with the new and traditional anthropometric indices, finding that only the ABSI independently showed an association with subclinical atherosclerosis (50).

The ABSI is a good estimator of central obesity and visceral adiposity (15) and is considered a non-invasive and simple tool, which can be easily applied to daily clinical practice. A significant association between ABSI and c-IMT has been shown in two previous studies (51). This relationship has also been observed between ABSI and the presence of carotid plaque (52). Similarly, this new anthropometric index has been related to an increase in diabetes mellitus, hypertension, and CV disease, outperforming WC and BMI in predicting CV and all-cause mortality (53, 54).

The CI hypnotized that subjects with less fat around the abdomen have a cylinder shape while those with more fat accumulation in the central area have a double-cone shape and was proposed as a predictor of corporal fat distribution and obesity (19, 21). Its value increases as a function of fat accumulation in the abdominal region of the body. In previous studies, the CI has been positively associated with insulin resistance, diabetes, hypertension, and dyslipidemia, although with exclusively older adult samples and divided by sex (55, 56). However, in a previous study, this anthropometric index was not associated with c-IMT (57). No other articles have studied this association.

In 2018, Park et al. suggested WWI as a novel simple anthropometric index of obesity to assess adiposity by standardizing WC by weight, showing a significant relationship with both CV mortality and morbidity (20). In contrast to BMI and WC, fat and muscle mass components could be better reflected (58). In subsequent studies, WWI has been shown to be a superior predictor of hypertension than WC and BMI and has been related to increased risk of diabetes (59, 60). In a prospective study with a cohort of 12,000 patients conducted earlier this year in southern China, higher levels of WWI were associated with an increased risk of CV and all-cause mortality (61). This is the first study to investigate the relationship between WWI and c-IMT.

In this study, ABSI, CI, and WWI were the anthropometric indices that were independently associated with pathological c-IMT (≥0.90 mm). These anthropometric indices have in common the result of adjusting WC for weight or BMI, being better indicators of central obesity and visceral adiposity than traditional anthropometric indices (14). Visceral adiposity is known to secrete vasoactive compounds that act as inflammatory mediators, such as protein C reactive or adipokines (e.g., interleukin-6, leptin, and TNF-α), possibly inducing changes in arterial structure and function (62–65), which may explain our findings. Inflammation plays an important role in all phases of atherosclerosis, including the initial activation of endothelial cells, progression, and, ultimately, its final complication of thrombosis (66).

Currently, there are no studies that provide the optimal cut-off values of novel anthropometric indices to detect pathological c-IMT (≥0.90 mm), so this study is the first to report such values.

This cross-sectional study has various limitations. First, this was an observational study; consequently, our results only suggest association and not cause–effect. Furthermore, all the individuals enrolled were from our province; therefore, the findings may not be appropriate for populations from other regions. On the other hand, most of the participants had a moderate or high risk of CV disease, and our results may not be applicable to lower-risk populations. Finally, age was statistically different between the two groups, and it is known that increasing the age also increases the cardiovascular risk in normoponderal subjects.

In conclusion, the results of our study, associated with the previous literature, indicate that the ABSI, CI, and WWI could be good predictors of subclinical atherosclerotic measured by c-IMT. Combinations of several anthropometric measures could further enhance CV risk prediction, and we suggest that its use should be implemented in daily clinical practice for the prevention and control of CV events.

## Data availability statement

The original contributions presented in the study are included in the article/supplementary material, further inquiries can be directed to the corresponding author (sergiorico@unex.es).

## Ethics statement

The studies involving human participants were reviewed and approved by Clinical Research and Ethics Committee (Ref. 047-2021) of Cáceres (Spain). The patients/participants provided their written informed consent to participate in this study.

## Author contributions

Conceptualization and writing—original draft preparation: CC-M, JC-G, SR-M, and JFSM-T. Methodology: CC-M, CS-B, JG-G, GE-S, FR-V, and ES-M. Validation: CC-M, JC-G, and SR-M. Formal analysis and writing—review and editing: JFSM-T and SR-M. Investigation: CC-M, JC-G, CS-B, JG-G, GE-S, FR-V, CF, and ES-M. Data curation: SR-M. Supervision: SR-M, JC-G, and JFSM-T. All authors contributed to the article and approved the submitted version.

## References

[1] Tsao CW Aday AW Almarzooq ZI Alonso A Beaton AZ Bittencourt MS. Heart disease and stroke statistics-2022 update: a report from the american heart association. Circulation. (2022) 145:e153–639. 10.1161/CIR.0000000000001052 35078371

[2] Timmis A Vardas P Townsend N Torbica A Katus H De Smedt D. European Society of Cardiology: cardiovascular disease statistics 2021. Eur Heart J. (2022) 43:716–99. 10.1093/eurheartj/ehab892 35016208

[3] Hansson GK. Inflammation, atherosclerosis, and coronary artery disease. N Engl J Med. (2005) 352:1685–95. 10.1056/NEJMra043430 15843671

[4] Lorenz MW Markus HS Bots ML Rosvall M Sitzer M. Prediction of clinical cardiovascular events with carotid intima-media thickness: a systematic review and meta-analysis. Circulation. (2007) 115:459–67. 10.1161/CIRCULATIONAHA.106.628875 17242284

[5] Naqvi TZ Lee M-S. Carotid intima-media thickness and plaque in cardiovascular risk assessment. JACC Cardiovasc Imaging. (2014) 7:1025–38. 10.1016/j.jcmg.2013.11.014 25051948

[6] Redberg RF Vogel RA Criqui MH Herrington DM Lima JAC Roman MJ. 34th Bethesda Conference: task force #3–what is the spectrum of current and emerging techniques for the noninvasive measurement of atherosclerosis? J Am Coll Cardiol. (2003) 41:1886–98. 10.1016/S0735-1097(03)00360-7 12798555

[7] Willeit P Tschiderer L Allara E Reuber K Seekircher L Gao L. Carotid intima-media thickness progression as surrogate marker for cardiovascular risk: meta-analysis of 119 clinical trials involving 100 667 patients. Circulation. (2020) 142:621–42. 10.1161/CIRCULATIONAHA.120.046361 32546049 PMC7115957

[8] Polak JF Pencina MJ Pencina KM O'Donnell CJ Wolf PA D'Agostino RB. Carotid-wall intima-media thickness and cardiovascular events. N Engl J Med. (2011) 365:213–21. 10.1056/NEJMoa1012592 21774709 PMC3153949

[9] Chambless LE Heiss G Folsom AR Rosamond W Szklo M Sharrett AR. Association of coronary heart disease incidence with carotid arterial wall thickness and major risk factors: the Atherosclerosis Risk in Communities (ARIC) Study, 1987-1993. Am J Epidemiol. (1997) 146:483–94. 10.1093/oxfordjournals.aje.a009302 9290509

[10] O'Leary DH Polak JF Kronmal RA Manolio TA Burke GL Wolfson Jr SK. Carotid-artery intima and media thickness as a risk factor for myocardial infarction and stroke in older adults. Cardiovascular Health Study Collaborative Research Group. N Engl J Med. (1999) 340:14–22. 10.1056/NEJM199901073400103 9878640

[11] Bots ML Hoes AW Koudstaal PJ Hofman A Grobbee DE. Common carotid intima-media thickness and risk of stroke and myocardial infarction: the Rotterdam Study. Circulation. (1997) 96:1432–7. 10.1161/01.CIR.96.5.1432 9315528

[12] Sinning C Wild PS Echevarria FMO Wilde S Schnabel R Lubos E. Sex differences in early carotid atherosclerosis (from the community-based Gutenberg-Heart Study). Am J Cardiol. (2011) 107:1841–7. 10.1016/j.amjcard.2011.02.318 21481827

[13] Jensen MD Ryan DH Apovian CM Ard JD Comuzzie AG Donato KA. 2013 AHA/ACC/TOS guideline for the management of overweight and obesity in adults: a report of the American College of Cardiology/American Heart Association Task Force on Practice Guidelines and the obesity society. J Am Coll Cardiol. (2013) 63:2985–3023. 10.1161/01.cir.0000437739.71477.ee 24239920

[14] Jayawardena R Ranasinghe P Ranathunga T Mathangasinghe Y Wasalathanththri S Hills AP. Novel anthropometric parameters to define obesity and obesity-related disease in adults: a systematic review. Nutr Rev. (2020) 78:498–513. 10.1093/nutrit/nuz078 31841153

[15] Krakauer NY Krakauer JC A. new body shape index predicts mortality hazard independently of body mass index. PLoS ONE. (2012) 7:e39504. 10.1371/journal.pone.0039504 22815707 PMC3399847

[16] Rico-Martín S Calderón-García JF Sánchez-Rey P Franco-Antonio C Martínez Alvarez M Sánchez Muñoz-Torrero JF. Effectiveness of body roundness index in predicting metabolic syndrome: a systematic review and meta-analysis. Obes Rev. (2020) 21:e13023. 10.1111/obr.13023 32267621

[17] Bergman RN Stefanovski D Buchanan TA Sumner AE Reynolds JC Sebring NG. Better index of body adiposity. Obesity (Silver Spring). (2011) 19:1083–9. 10.1038/oby.2011.38 21372804 PMC3275633

[18] Gómez-Ambrosi J Silva C Catalán V Rodríguez A Galofré JC Escalada J. Clinical usefulness of a new equation for estimating body fat. Diabetes Care. (2012) 35:383–8. 10.2337/dc11-1334 22179957 PMC3263863

[19] Guerrero-Romero F Rodriguez-Morán M. Abdominal volume index. An anthropometry-based index for estimation of obesity is strongly related to impaired glucose tolerance and type 2 diabetes mellitus. Arch Med Res. (2003) 34:428–32. 10.1016/S0188-4409(03)00073-0 14602511

[20] Park Y Kim NH Kwon TY Kim SG. A novel adiposity index as an integrated predictor of cardiometabolic disease morbidity and mortality. Sci Rep. (2018) 8:16753. 10.1038/s41598-018-35073-4 30425288 PMC6233180

[21] Valdez R. A simple model-based index of abdominal adiposity. J Clin Epidemiol. (1991) 44:955–6. 10.1016/0895-4356(91)90059-I 1890438

[22] Williams B Mancia G Spiering W Agabiti Rosei E Azizi M Burnier M. 2018 Practice guidelines for the management of arterial hypertension of the european society of hypertension and the european society of cardiology: ESH/ESC task force for the management of arterial hypertension. J Hypertens. (2018) 36:2284–309. 10.1097/HJH.0000000000001961 30379783

[23] Salas-Salvadó J Rubio MA Barbany M Moreno B Grupo Colaborativo de la SEEDO. SEEDO 2007 Consensus for the evaluation of overweight and obesity and the establishment of therapeutic intervention criteria. Med Clin (Barc). (2007) 128:184–96. 10.1016/S0025-7753(07)72531-9 17298782

[24] Touboul P-J Hennerici MG Meairs S Adams H Amarenco P Bornstein N. Mannheim carotid intima-media thickness and plaque consensus (2004-2006-2011). An update on behalf of the advisory board of the 3rd, 4th and 5th watching the risk symposia, at the 13th, 15th and 20th European Stroke Conferences, Mannheim, Germany, 2004, Brussels, Belgium, 2006, and Hamburg, Germany, 2011. Cerebrovasc Dis. (2012) 34:290–6. 10.1159/000343145 23128470 PMC3760791

[25] Vlachopoulos C Xaplanteris P Aboyans V Brodmann M Cífková R Cosentino F. The role of vascular biomarkers for primary and secondary prevention a position paper from the European Society of Cardiology Working Group on peripheral circulation: Endorsed by the Association for Research into Arterial Structure and Physiology (ARTERY) Society. Atherosclerosis. (2015) 241:507–32. 10.1016/j.atherosclerosis.2015.05.007 26117398

[26] Herder M Johnsen SH Arne A Mathiesen EB. Risk factors for progression of carotid intima-media thickness and total plaque area: a 13-year follow-up study: the Tromsø study. Stroke. (2012) 43:1818–23. 10.1161/STROKEAHA.111.646596 22550052

[27] Inaba Y Chen JA Bergmann SR. Carotid plaque, compared with carotid intima-media thickness, more accurately predicts coronary artery disease events: a meta-analysis. Atherosclerosis. (2011) 220:128–33. 10.1016/j.atherosclerosis.2011.06.044 21764060

[28] Mach F Baigent C Catapano AL Koskinas KC Casula M Badimon L. 2019 ESC/EAS Guidelines for the management of dyslipidaemias: lipid modification to reduce cardiovascular risk. Eur Heart J. (2020) 41:111–88. 10.1093/eurheartj/ehz455 31504418

[29] Baber U Mehran R Sartori S Schoos MM Sillesen H Muntendam P. Prevalence, impact, and predictive value of detecting subclinical coronary and carotid atherosclerosis in asymptomatic adults: the BioImage study. J Am Coll Cardiol. (2015) 65:1065–74. 10.1016/j.jacc.2015.01.017 25790876

[30] McDermott MM Kramer CM Tian L Carr J Guralnik JM Polonsky T. Plaque composition in the proximal superficial femoral artery and peripheral artery disease events. JACC Cardiovasc Imaging. (2016) 10:1003–12. 10.1016/j.jcmg.2016.08.012 27838307 PMC5701810

[31] Sillesen H Sartori S Sandholt B Baber U Mehran R Fuster V. Carotid plaque thickness and carotid plaque burden predict future cardiovascular events in asymptomatic adult Americans. Eur Heart J Cardiovasc Imaging. (2018) 19:1042–50. 10.1093/ehjci/jex239 29059296

[32] Yeboah J McClelland RL Polonsky TS Burke GL Sibley CT O'Leary D. Comparison of novel risk markers for improvement in cardiovascular risk assessment in intermediate-risk individuals. JAMA. (2012) 308:788–95. 10.1001/jama.2012.9624 22910756 PMC4141475

[33] den Ruijter HM Peters SAE Anderson Todd J Britton AR Dekker JM Eijkemans MJ. Common carotid intima-media thickness measurements in cardiovascular risk prediction: a meta-analysis. JAMA. (2012) 308:796–803. 10.1001/jama.2012.9630 22910757

[34] Eigenbrodt ML Bursac Z Tracy RE Mehta JL Rose KM Couper DJ. B-mode ultrasound common carotid artery intima-media thickness and external diameter: cross-sectional and longitudinal associations with carotid atherosclerosis in a large population sample. Cardiovasc Ultrasound. (2008) 6:10. 10.1186/1476-7120-6-10 18321381 PMC2277382

[35] Xie W Liu J Wang W Wang M Li Y Sun J. Five-year change in systolic blood pressure is independently associated with carotid atherosclerosis progression: a population-based cohort study. Hypertens Res. (2014) 37:960–5. 10.1038/hr.2014.93 24804610

[36] Zureik M Ducimetière P Touboul PJ Courbon D Bonithon-Kopp C Berr C. Common carotid intima-media thickness predicts occurrence of carotid atherosclerotic plaques: longitudinal results from the Aging Vascular Study (EVA) study. Arterioscler Thromb Vasc Biol. (2000) 20:1622–9. 10.1161/01.ATV.20.6.1622 10845881

[37] Prati P Vanuzzo D Casaroli M Bader G Mos L Pilotto L. Determinants of carotid plaque occurrence A long-term prospective population study: the San Daniele Project. Cerebrovasc Dis. (2006) 22:416–22. 10.1159/000094993 16912475

[38] Spence JD. Measurement of intima-media thickness vs. carotid plaque: uses in patient care, genetic research and evaluation of new therapies. Int J Stroke. (2006) 1:216–21. 10.1111/j.1747-4949.2006.00068.x 18706019

[39] Finn A Kolodgie FD Virmani R. Correlation between carotid intimal/medial thickness and atherosclerosis: a point of view from pathology. Arterioscler Thromb Vasc Biol. (2009) 30:177–81. 10.1161/ATVBAHA.108.173609 19679833

[40] Bis JC Kavousi M Franceschini N Isaacs A Abecasis GR Schminke U. Meta-analysis of genome-wide association studies from the CHARGE consortium identifies common variants associated with carotid intima media thickness and plaque. Nat Genet. (2011) 43:940–7. 21909108 10.1038/ng.920 PMC3257519

[41] Franceschini N Giambartolomei C de Vries PS Finan C Bis JC Huntley RP. GWAS and colocalization analyses implicate carotid intima-media thickness and carotid plaque loci in cardiovascular outcomes. Nat Commun. (2018) 9:5141. 10.1038/s41467-018-07340-5 30510157 PMC6277418

[42] Nambi V Chambless L Folsom AR Max H Hu Y Mosley T. Carotid intima-media thickness and presence or absence of plaque improves prediction of coronary heart disease risk: the ARIC (Atherosclerosis Risk In Communities) study. J Am Coll Cardiol. (2010) 55:1600–7. 10.1016/j.jacc.2009.11.075 20378078 PMC2862308

[43] Nicolaides AN Panayiotou AG Maura G Tyllis T Bond D Georgiou N. Arterial ultrasound testing to predict atherosclerotic cardiovascular events. J Am Coll Cardiol. (2022) 79:1969–82. 10.1016/j.jacc.2022.03.352 35589158

[44] Cameron AJ Magliano DJ Shaw JE Zimmet PZ Carstensen B Alberti KGM. The influence of hip circumference on the relationship between abdominal obesity and mortality. Int J Epidemiol. (2012) 41:484–94. 10.1093/ije/dyr198 22266094 PMC3324456

[45] Mottillo S Filion KB Genest J Joseph L Pilote L Poirier P. The metabolic syndrome and cardiovascular risk a systematic review and meta-analysis. J Am Coll Cardiol. (2010) 56:1113–32. 10.1016/j.jacc.2010.05.034 20863953

[46] Ortega FB Sui X Lavie CJ Blair SN. Body mass index, the most widely used but also widely criticized index: would a criterion standard measure of total body fat be a better predictor of cardiovascular disease mortality? Mayo Clin Proc. (2016) 91:443–55. 10.1016/j.mayocp.2016.01.008 26948431 PMC4821662

[47] Visseren FLJ Mach F Smulders YM Carballo D Koskinas KC Bäck M. 2021 ESC guidelines on cardiovascular disease prevention in clinical practice. Eur Heart J. (2021) 42:3227–337. 10.1093/eurheartj/ehab484 34458905

[48] Bray GA Heisel WE Afshin A Jensen MD Dietz WH Long M. The science of obesity management: an endocrine society scientific statement. Endocr Rev. (2018) 39:79–132. 10.1210/er.2017-00253 29518206 PMC5888222

[49] Nevill AM Stewart AD Olds T Holder R. Relationship between adiposity and body size reveals limitations of BMI. Am J Phys Anthropol. (2006) 129:151–6. 10.1002/ajpa.20262 16270304

[50] Costo-Muriel C Calderón-Garcia JF Rico-Martin S Sánchez-Bacaicoa C Escudero-Sánchez G Galán-González J. Association of subclinical carotid atherosclerosis assessed by high-resolution ultrasound with traditional and novel anthropometric indices. Curr Probl Cardiol. (2022) 48:101574. 10.1016/j.cpcardiol.2022.101574 36584728

[51] Gomez-Marcos MA Gomez-Sanchez L Patino-Alonso MC Recio-Rodriguez JI Gomez-Sanchez M Rigo F. A body shape index and vascular structure and function in Spanish adults (MARK study): a cross-sectional study. Medicine (Baltimore). (2018) 97:e13299. 10.1097/MD.0000000000013299 30461641 PMC6392544

[52] Ma X Chen L Hu W He L. Association between a body shape index and subclinical carotid atherosclerosis in population free of cardiovascular and cerebrovascular diseases. J Atheroscler Thromb. (2021) 29:1140–52. 10.5551/jat.62988 34483222 PMC9371761

[53] Calderón-Garcia JF Roncero-Martín R Rico-Martín S De Nicolás-Jiménez JM López-Espuela F Santano-Mogena E. Effectiveness of body roundness index (BRI) and a body shape index (ABSI) in predicting hypertension: a systematic review and meta-analysis of observational studies. Int J Environ Res Public Health. (2021) 18:ijerph182111607. 10.3390/ijerph182111607 34770120 PMC8582804

[54] Ji M Zhang S An R. Effectiveness of A Body Shape Index (ABSI) in predicting chronic diseases and mortality: a systematic review and meta-analysis. Obes Rev. (2018) 19:737–59. 10.1111/obr.12666 29349876

[55] Andrade MD Freitas MCP de Sakumoto AM Pappiani C Andrade SC de Vieira VL. Association of the conicity index with diabetes and hypertension in Brazilian women. Arch Endocrinol Metab. (2016) 60:436–42. 10.1590/2359-3997000000187 27812606 PMC10118640

[56] Nkwana MR Monyeki KD Lebelo SL. Body roundness index, a body shape index, conicity index, and their association with nutritional status and cardiovascular risk factors in South African rural young adults. Int J Environ Res Public Health. (2021) 18:ijerph18010281. 10.3390/ijerph18010281 33401502 PMC7795753

[57] Sánchez E Sánchez M Àngels B Rius F Torres G Purroy F. Are obesity indices useful for detecting subclinical atheromatosis in a middle-aged population? Obes Facts. (2020) 13:29–39. 10.1159/000502696 31968341 PMC7098313

[58] Kim NH Park Y Kim NH Kim SG. Weight-adjusted waist index reflects fat and muscle mass in the opposite direction in older adults. Age Ageing. (2021) 50:780–6. 10.1093/ageing/afaa208 33035293

[59] Li Q Qie R Qin P Zhang D Guo C Zhou Q. Association of weight-adjusted-waist index with incident hypertension: the rural Chinese cohort study. Nutr Metab Cardiovasc Dis. (2020) 30:1732–41. 10.1016/j.numecd.2020.05.033 32624344

[60] Liu Y Liu X Zhang S Qibo Z Fu X Chen H. Association of anthropometric indices with the development of diabetes among hypertensive patients in china: a cohort study. Front Endocrinol (Lausanne). (2021) 12:736077. 10.3389/fendo.2021.736077 34675879 PMC8525507

[61] Ding C Shi Y Li J Li M Hu L Rao J. Association of weight-adjusted-waist index with all-cause and cardiovascular mortality in China: a prospective cohort study. Nutr Metab Cardiovasc Dis. (2022) 32:1210–7. 10.1016/j.numecd.2022.01.033 35277327

[62] Singhal A Farooqi IS Cole TJ O'Rahilly S Fewtrell M Kattenhorn M. Influence of leptin on arterial distensibility: a novel link between obesity and cardiovascular disease? Circulation. (2002) 106:1919–24. 10.1161/01.CIR.0000033219.24717.52 12370213

[63] Rocha VZ Libby P. The multiple facets of the fat tissue. Thyroid. (2008) 18:175–83. 10.1089/thy.2007.0296 18279018

[64] Kim K Valentine RJ Shin Y Gong K. Associations of visceral adiposity and exercise participation with C-reactive protein, insulin resistance, and endothelial dysfunction in Korean healthy adults. Metabolism. (2008) 57:1181–9. 10.1016/j.metabol.2008.04.009 18702942

[65] Tsuriya D Morita H Morioka T Takahashi N Ito T Oki Y. Significant correlation between visceral adiposity and high-sensitivity C-reactive protein (hs-CRP) in Japanese subjects. Intern Med. (2011) 50:2767–73. 10.2169/internalmedicine.50.5908 22082888

[66] Libby P Ridker PM Maseri A. Inflammation and atherosclerosis. Circulation. (2002) 105:1135–43. 10.1161/hc0902.104353 11877368

